# Examining the acceptability and feasibility of the Compassionate Mindful Resilience (CMR) programme in adults living with chronic kidney disease: the COSMIC study findings

**DOI:** 10.1186/s12882-024-03473-6

**Published:** 2024-01-31

**Authors:** Anna Wilson, Claire Carswell, Clare McKeaveney, Karen Atkinson, Stephanie Burton, Clare McVeigh, Lisa Graham-Wisener, Erika Jääskeläinen, William Johnston, Daniel O’Rourke, Joanne Reid, Soham Rej, Ian Walsh, Helen Noble

**Affiliations:** 1 School of Nursing and Midwifery, Queen’s University Belfast, Belfast, UK; 2 Department of Health Sciences, University of York, York, UK; 3 MindfulnessUK, Tauton, UK; 4 School of Psychology, Queen’s University Belfast, Belfast, UK; 5 Research Unit of Population Health, University of Oulu, Oulu, Finland; 6 Patient and Carer Education Partnership, School of Nursing and Midwifery, Queen’s University Belfast, Belfast, UK; 7 Department of Psychiatry, McGill University, Montreal, Canada; 8 School of Medicine, Dentistry and Biomedical Sciences, Queen’s University Belfast, Belfast Northern, Ireland; 9 Knightsbridge Healthcare Group, Belfast, UK; 10 Institute of Psychosexual Medicine, London, UK

**Keywords:** Feasibility, Kidney disease, Kidney transplant, Mindfulness, Wellbeing

## Abstract

**Background:**

Individuals with chronic kidney disease experience difficult physical and psychological symptoms, that impact quality of life, and are at increased risk of anxiety and depression. Access to specialist psychological support is limited. This study aimed to support a new service development project, in collaboration with Kidney Care UK, to implement the Compassionate Mindful Resilience (CMR) programme, developed by MindfulnessUK, which provides accessible mindfulness techniques and practices to enhance compassion and resilience, and explore its feasibility for people living with stage 4 or 5 kidney disease and transplant.

**Methods:**

A multi-method feasibility design was utilised. Participants over 18 years, from the UK, with stage 4 or 5 kidney disease or post-transplant, and who were not currently undergoing psychotherapy, were recruited to the four-week CMR programme. Data was collected at baseline, post-intervention and three-months post to measure anxiety, depression, self-compassion, mental wellbeing, resilience, and mindfulness. The acceptability of the intervention for a kidney disease population was explored through qualitative interviews with participants, and the Mindfulness Teacher.

**Results:**

In total, 75 participants were recruited to the study, with 65 completing the CMR programme. The majority were female (66.2%) and post-transplant (63.1%). Analysis of completed outcome measures at baseline and post-intervention timepoints (*n* = 61), and three-months post intervention (*n* = 45) revealed significant improvements in participant’s levels of anxiety (*p* < .001) and depression (*p* < .001), self-compassion (*p* = .005), mental wellbeing (*p* < .001), resilience (*p*.001), and mindfulness (*p* < .001).

Thematic analysis of interviews with participants (*n* = 19) and Mindfulness Teacher (*n* = 1) generated three themes (and nine-subthemes); experiences of the CMR programme that facilitated subjective benefit, participants lived and shared experiences, and practicalities of programme participation. All participants interviewed reported that they found programme participation to be beneficial.

**Conclusion:**

The findings suggest that the CMR programme has the potential to improve psychological outcomes among people with chronic kidney disease. Future randomized controlled trials are required to further test its effectiveness.

**Supplementary Information:**

The online version contains supplementary material available at 10.1186/s12882-024-03473-6.

## Introduction

Individuals with chronic kidney disease often experience challenging physical and psychological symptoms, leading to a diminished quality of life [1], and are at an increased risk of anxiety [2], and depression [3, 4], with between 12 and 52% diagnosed with anxiety [5], and approximately 20% diagnosed with depression [6]. Access to specialist psychological or social support is limited, as highlighted by the recent UK renal psychosocial workforce report [7]. The COVID-19 pandemic has further exacerbated the situation, with people with kidney disease continuing to experience heightened risk factors for serious illness and mortality due to their clinically extremely vulnerable status [8], and prolonged periods of shielding and social distancing [9]. Consequently, there has been an increase in psychological distress for many people living with kidney disease [10], with an increase in anxiety and depression [11], highlighting the need for effective psychosocial support systems and interventions.

Mindfulness is the ability to focus attention on the present moment, and promotes acceptance of thoughts and feelings without judgement [12]. In recent years, mindfulness interventions, such as the Mindfulness-Based Stress Reduction (MBSR) programme, have gained popularity as an effective stress-reduction technique applicable across a range of settings [12, 13]. Developed by Professor Jon Kabat-Zinn at the University of Massachusetts Medical Center, the eight-week MBSR programme focuses on an individual’s attention to internal and external stimuli to remain in the present moment and reduce disruptive thoughts [14]. Mindfulness interventions have been successfully implemented for people with a range of chronic conditions including fibromyalgia, type 2 diabetes, and cardiovascular disease, and have shown positive outcomes, such as improved wellbeing and quality of life [15], and emerging research has highlighted how integrating compassion into mindfulness programmes can improve psychological and physiological outcomes for chronic illness populations [16]. However, most evidence for mindfulness interventions require several months of commitment from participants and are delivered in person, a significant barrier to engagement for people with chronic illness since the COVID-19 pandemic. There is a lack of research on the effectiveness of brief mindfulness interventions that are delivered remotely for people with kidney disease [17].

To address this gap, a new service development project was initiated in partnership with the UK’s leading patient support charity, Kidney Care UK [18]. The project implemented the Compassionate Mindful Resilience (CMR) programme, developed by MindfulnessUK [19], and aimed to explore the acceptability and feasibility of the intervention for people living with stage 4 or 5 chronic kidney disease or who have received a kidney transplant. It was hypothesized that the CMR programme is a feasible and acceptable intervention for this patient group.

### Intervention

The CMR programme was developed by Karen Atkinson, executive committee member for the British Association of Mindfulness Based Approaches [20] and MindfulnessUK, to provide accessible mindfulness and compassion practices. It consists of two-hour sessions over four consecutive weeks, delivered in an online group setting, focusing on enhancing self-compassion and resilience through evidence-based practices such as body scan, compassionate movement, and meditation. The programme was developed in response to a need for accessible stress management techniques and draws on Buddhist and Yoga philosophy blended with current psychoeducational intervention practices [21]. Home practices are provided but are optional. Participants were provided with a Resource Pack which provided materials and practices to support each session and a diary to record activities, along with three audio meditations: Compassionate Body Scan, Affectionate Breathing Practice and Compassionate Movement Meditation. The first programme was delivered by HN, an experienced nephrology nurse, and subsequent sessions were delivered by Dr Michele Kavanagh, Consultant Clinical Psychologist, both of whom trained with MindfulnessUK in delivering the CMR programme.

## Methods

### Design

A single-group multi-methods feasibility study was used, including a qualitative exploration of acceptability. The protocol for the study has been published [22].

### Recruitment

Kidney Care UK promoted the CMR programme and details of the study via their e-newsletter, website and Facebook patient group in May and September 2022. Potential participants were directed to complete an online form to register their interest, and to confirm they understood and met the eligibility criteria for the study.

#### Eligibility criteria:

- Over 18 years
- Currently living in the UK
- In stage 4 or 5 kidney disease or have received a kidney transplant
- Capacity to provide informed consent.
- Not currently undergoing psychotherapy

#### Exclusion criteria

- The CMR programme is not recommended for individuals experiencing severe anxiety, depression, mental illness, addiction, recent bereavement, or a traumatic life event. Participants were advised of this prior to registering for the study, and any ongoing concerns were explored during the assessment prior to commencing the programme.

Potential participants who met the eligibility criteria were sent a participant information sheet and a consent form via email. Participants who returned completed consent forms were referred to the Mindfulness Teacher for a standardised formal assessment via telephone, which had been adapted in consultation with the Expert Advisory Group, including Public and Patient Involvement (PPI) representation, to ensure the CMR programme was appropriate for a kidney disease population and to identify if additional support was required for programme participation. Participants were then allocated to a CMR group at a suitable time and date, and were sent the questionnaires and instructions for completion two weeks prior to commencing the programme.

### Sample size

Using G*Power software, a calculation was conducted to determine the appropriate sample size needed to detect a minimal clinically important difference [23] in the Generalised Anxiety Disorder questionnaire (GAD-7) [24], as this is the proposed primary clinical outcome in a future definitive trial. The calculation was based on study data that assessed a complex intervention for managing anxiety and depression in individuals with chronic disease [25]. A sample size of 75 participants was deemed necessary to achieve 95% power in detecting a clinically important difference in the primary outcome measure allowing for a 20% attrition rate [26]. Therefore, in order to evaluate the feasibility of recruiting participants into an adequately powered study, the target sample size was 75 participants.

### Data collection

Data collection took place between May 2022 and February 2023. Participants were asked to complete measurements at three timepoints; a baseline measurement approximately two weeks before commencing the programme, and follow-up measurements on completion of the programme, and at three months post-intervention. Demographic information was collected alongside the baseline measurement, including age, gender, socioeconomic status, relationship status, education level, and CKD status. The standardised measures were implemented via online survey using Qualtrics software [27] at the three timepoints. Participant data was pseudonymised prior to review and analysis by the wider research team.

### Clinical outcomes

The clinical outcomes of interest were anxiety, depression, self-compassion, mindfulness, wellbeing, and resilience. The following measures were used to assess the feasibility of data collection, determined by the proportion of missing data.

- Anxiety was measured using the Generalised Anxiety Disorder Assessment (GAD-7), a brief self-report scale consisting of seven items based on the DSM-IV criteria for generalized anxiety disorder [24].
- Depression was assessed using the Patient Health Questionnaire (PHQ-9), a brief self-report scale consisting of nine items based on the DSM-IV criteria for major depressive disorder [28].
- Self-compassion was measured using the shortened 12-item version of the Self-Compassion Scale (SCS-SF) [29].
- Mindfulness was assessed using the Five Facet Mindfulness Questionnaire (FFMQ), a self-report measure consisting of 39 items that measure five facets of mindfulness and self-awareness [30].
- Wellbeing was measured using the Short Warwick-Edinburgh Mental Well-Being Scale (SWEMWBS), a validated and globally used scale consisting of seven positively worded items related to psychological and eudemonic wellbeing [31].
- Resilience was assessed using the Mental Toughness Questionnaire (MTQ48), a widely used and researched self-report measure consisting of four core components (challenge, commitment, control, and confidence) [32].

### Data analysis

The Statistical Package for the Social Sciences (SPSS v.28) [33] was used to assist with data analysis. To explore the potential effectiveness of the intervention, a repeated measures t-test was conducted to compare the mean scores of the outcome measures before and after the intervention. A repeated measures ANOVA was used to compare the difference in mean scores across the three data collection timepoints (baseline, post, and three months post). Data analysis was undertaken by Research Fellow CC and reviewed by Chief Investigator HN and Research Assistant AW.

### Qualitative interviews

#### Recruitment

Participants who completed the four-session CMR programme were invited to participate in a qualitative interview to explore participants’ experiences and the acceptability of the intervention. Participants who expressed interest were sent a participant information sheet and consent form via email to complete and return. A purposive sampling strategy was utilised to include a range of participants and representation from each of the eight CMR programme groups. The Mindfulness Teacher who taught seven of the eight groups was also interviewed.

### Sample size

In qualitative research, there are no specific guidelines to determine the ideal sample size to reach data saturation [34]. The study aimed to recruit 20 participants, to include the Mindfulness teacher. Guest et al. suggest that a sample size of 12 is appropriate for data saturation [35], however recruitment continued until the researchers deemed data saturation had been met.

### Data collection

The semi-structured interviews were informed by the RE-AIM QuEST framework [36], which proposes open-ended questions across five dimensions of Reach, Effectiveness, Adoption, Implementation, and Maintenance to guide evaluation of the intervention. Study participants received the intervention between June and October 2022, and interviews took place within two to seven weeks of programme completion. The interviews were conducted via the Zoom online meeting platform [37] at a time and date convenient for participants. The Research Assistant AW conducted the interviews, which were reviewed by the Chief Investigator HN. Participant data was pseudonymised prior to review and analysis by the wider research team. The interviews ranged from 20 to 60 min in duration.

### Data analysis

The semi-structured interviews were recorded and transcribed verbatim by a professional transcriber then analysed thematically utilising NVivo qualitative analysis software v.12 [38]. An inductive approach was utilized for thematic analysis, following a six-stage process: familiarisation with data, generating initial codes, searching for themes, reviewing themes, defining and naming themes, and producing the reported themes [39]. The interviews were coded and analysed by the Research Assistant AW and Chief Investigator HN, both of whom have extensive experience in qualitative research methodology and were reviewed by the research team. Data saturation was achieved after 20 interviews. Member checking was not undertaken as the research team did not wish to cause additional burden to the participants.

## Results

### Recruitment

**Fig. 1:**
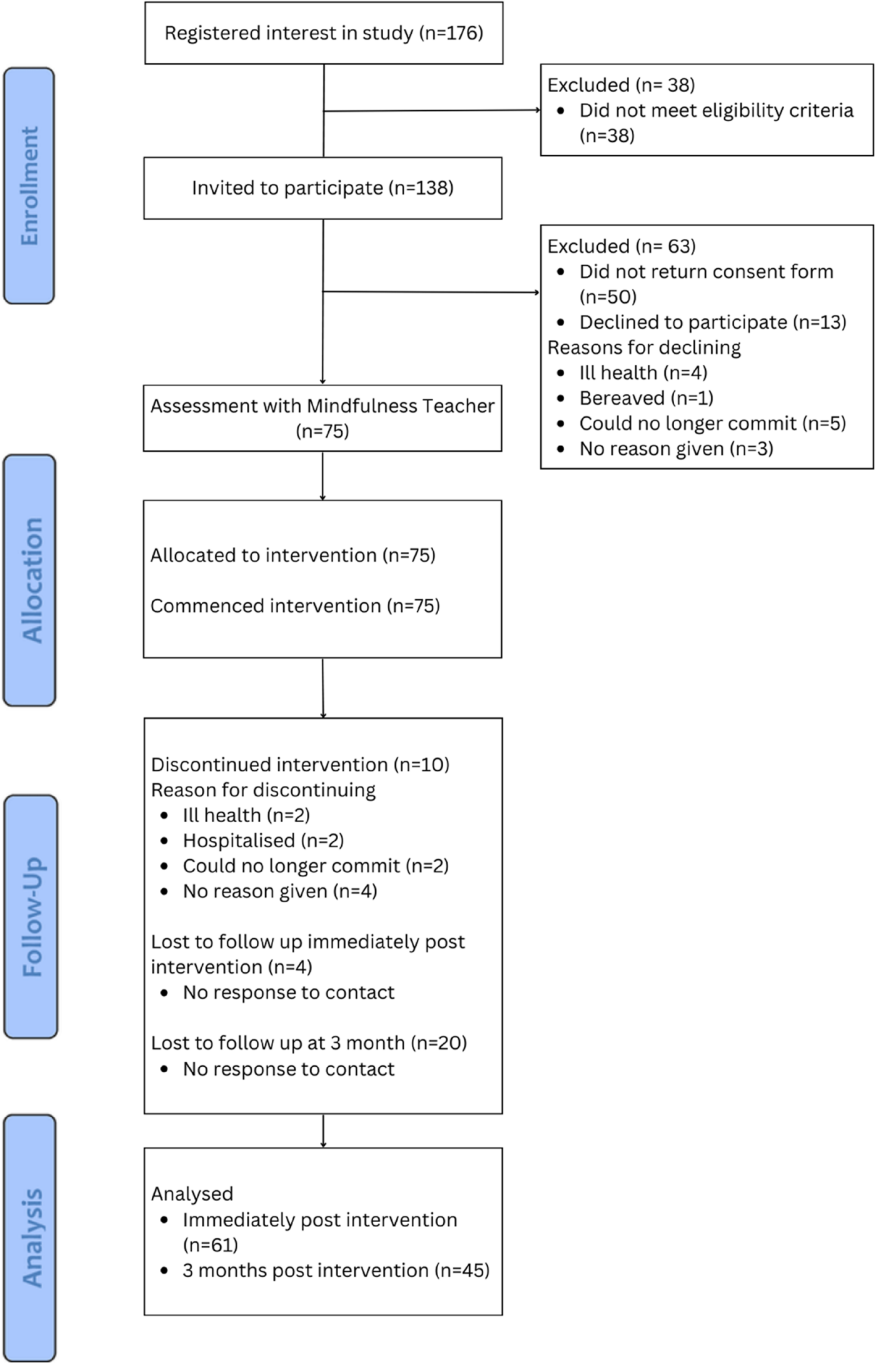
Recruitment flow diagram

Out of 176 people who registered their interest in the study, 38 (21.6%) did not meet the eligibility criteria. Participant information sheets and consent forms were sent to 138 people, and 50 (36.2%) did not return a completed consent form. The target sample of 75 participants was reached within four weeks of recruitment. Participants were referred to the Mindfulness Teacher to undertake the standardised formal assessment, prior to commencing the CMR programme. An overview of the flow of participants through the study can be seen in the recruitment flow diagram in Fig. 1.

### Retention and adherence

The attrition rate across the four-week duration of the CMR programme was 13.3% (*n* = 10). Reasons for withdrawal included participant ill health, unexpected hospitalisation, inability to commit to the duration of the programme and four participants discontinued the programme with no reason given. Participants who withdrew did not provide consent for their baseline data to be used in analysis. The remaining 65 participants attended three or four sessions of the programme, with a minimum threshold of attendance at three sessions to complete the programme.

Additionally, 5.3% (*n* = 4) were lost to follow-up during immediate post-intervention data collection, and a further 21.3% (*n* = 16) of participants were lost to follow-up at the three-month data collection timepoint. The overall attrition rate was 40% (*n* = 30) from baseline to study end.

### Participant characteristics

Of the 65 participants who completed the CMR programme, 66.2% (*n* = 43) identified as female, with a mean age of 56.2 years. The majority identified as English, Welsh, Scottish, Northern Irish, or British (64.6%, *n* = 42), married (66.2%, *n* = 43), post-transplant (63.1%, *n* = 41), had a level 4 + education (degree, higher degree, professional qualification) (72.3%, *n* = 47) and lived in England (81.54%, *n* = 53). The mean time since diagnosis was 20.26 years. An overview of participant characteristics is provided in Table 1.

**Table 1:** CMR participant characteristics

**Characteristic**	**Participants analysed (*n* = 65)**
**Age (years)**	
n (%)	65 (100%)
Mean (SD)	56.2 (9.42)
**Gender, n (%)**	
Number with data	65 (100%)
Female	43 (66.2%)
Male	22 (33.8%)
**Ethnicity, n (%)**	
Number with data	65 (100%)
Black, African, Caribbean or Black British—African	3 (4.6%)
Asian or Asian British—Any other Asian background	1 (1.5%)
White—Any other White background	7 (10.8%)
Arab	1 (1.5%)
Black, African, Caribbean or Black British—Caribbean	2 (3.1%)
Asian or Asian British—Chinese	1 (1.5%)
White—English, Welsh, Scottish, Northern Irish or British	42 (64.6%)
Asian or Asian British—Indian	3 (4.6%)
White—Irish	3 (4.6%)
Asian or Asian British—Pakistani	1 (1.5%)
Mixed or multiple ethnic groups—White and Black African	1 (1.5%)
**Marital status, n (%)**	
Number with data	65 (100%)
Divorced	9 (13.8%)
Married	43 (66.2%)
Widowed	2 (3.1%)
Single	11 (16.9%)
**Highest educational level, n (%)**	
Number with data	65 (100%)
Level 1	3 (4.6%)
Level 2	4 (6.2%)
Level 3	7 (10.8%)
Level 4 +	47 (72.3%)
No qualifications	1 (1.5%)
Other	3 (4.6%)
**Years since diagnosis (years)**	
n (%)	65 (100%)
Mean (SD)	20.26 (14.1)
**Stage of CKD, n (%)**	
Number with data	65 (100%)
Post-transplant	41 (63.1%)
Stage 5	15 (23.1%)
Stage 4	9 (13.8%)
**Region of UK, n (%)**	
Number with data	65 (100%)
England	53 (81.54%)
Scotland	6 (9.23%)
Wales	2 (3.08%)
Northern Ireland	4 (6.15%)

### Clinical outcome measures

Completion rates differed across outcome measures. At baseline 100% of participants (*n* = 65) completed the GAD-7, PHQ-9, and SCS-F, 98% of participants (*n* = 64) completed the FFMQ and SWEMWBS, while 95% of participants (*n* = 62) completed the MTQ48. An overview of the response rates and mean scores across the three time points are shown in Table 2.

**Table 2:** Outcome measures

	Baseline *n* = 65	Post-Intervention *n* = 61	Three-month Follow-up *n* = 45
Anxiety (GAD-7)			
n (%)	65 (100%)	61 (100%)	45 (100%)
Mean (SD)	7.3 (4.9)	5.8 (4.9)	4.4 (3.5)
Depression (PHQ-9)			
n (%)	65 (100%)	61 (100%)	45 (100%)
Mean (SD)	9.3 (5.7)	7.2 (5.6)	5.4 (4.6)
Self-Compassion (SCS-SF)			
n (%)	65 (100%)	60 (98%)	45 (100%)
Mean (SD)	2.9 (0.6)	3.3 (0.6)	3.3 (0.7)
Mindfulness (FFMQ)			
n (%)	64 (98%)	58 (95%)	44 (97%)
Mean (SD)	117.6 (17)	127.4 (17.4)	133.9 (19.5)
Mental Wellbeing (SWEMWBS)			
n (%)	64 (98%)	58 (95%)	44 (97%)
Mean (SD)	20.5 (2.9)	22.1 (3.9)	23.3 (3.6)
Resilience (MTQ48)			
n (%)	62 (95%)	55 (90%)	38 (93%)
Mean (SD	3.8 (1.7)	4.5 (1.8)	4.6 (1.9)

### Change in outcome measures from baseline

Table 2 outlines the mean scores for each of the scales across the follow-up period. There was a reduction in anxiety and depression as measured by the GAD-7 and PHQ-9, from baseline to post-intervention, and from baseline to three-month follow-up. There was an increase in self-compassion (SCSF), mindfulness (FFMQ), mental-wellbeing (SWEMWBS) and resilience (MTQ-48). While this is a feasibility study and effectiveness of the intervention is not being evaluated, statistical significance was explored to assess the appropriateness of the outcome measures for a definitive trial. Table 3 outlines the results of repeated measures t-tests, demonstrating the mean change in scores across the follow-up time points compared to baseline.

**Table 3:** Mean change in scores across follow-up time points

**Paired differences**			
	**Mean (SD)**	**95% CI**	***P* (one-sided)**
GAD-7—Baseline to post-intervention	1.5 (4.9)	0.2, 2.7	.011*
GAD-7—Baseline to 3-month follow-up	2.1 (3.8)	0.9, 3.2	< .001*
PHQ-9—Baseline to post-intervention	2.3 (4.9)	1.1, 3.6	< .001*
PHQ-9—Baseline to 3-month follow-up	2.9 (5.1)	1.3, 4.5	< .001*
SCS-SF—Baseline to post-intervention	-0.4 (0.7)	-0.6, -0.3	< .001*
SCS-SF—Baseline to 3-month follow-up	-0.3 (0.8)	-0.6, 0.1	.005*
FFMQ—Baseline to post-intervention	-9.3 (16.6)	-13.6, -4.9	< .001*
FFMQ—Baseline to 3-month follow-up	-16.1 (19.6)	-22.1, -10.1	< .001*
SWEMWBS—Baseline to post-intervention	-1.7 (3.3)	-2.6, -0.8	< .001*
SWEMWBS—Baseline to 3-month follow-up	-2.4 (3.7)	-3.6, -1.2	< .001*
MTQ-48—Baseline to post-intervention	-0.7 (1.5)	-1.1, -0.3	< .001*
MTQ-48—Baseline to 3-month follow-up	-1 (1.5)	-1.5, -0.5	< .001*

^*^statistically significant

### Intervention adherence

The CMR intervention provides participants with the knowledge and techniques to engage in mindfulness practices at home, following completion of the four-week programme, and the proportion of participants who continued to engage in mindfulness practices was explored at the three-month follow-up timepoint. 89% of participants who responded to the questionnaire at three-months post intervention had continued to engage in mindfulness practices, with most respondents engaging in these daily (46%) or several times a week (23%). An overview of participants’ ongoing practices can be seen in Table 4.

**Table 4:** Participants continuing to practice mindfulness

	**Three-month follow-up (*n* = 45)**
Continuing to practice?	
Number with data	45 (100%)
Yes	40 (89%)
No	5 (11%)
If yes, how often?	
Number with data	39 (87%)
Daily	18 (46%)
Several times a week	11 (28%)
Weekly	9 (23%)
Occasionally	1 (3%)

### Qualitative interviews

Of the 65 participants invited to participate in the interview, 19 participants were interviewed, representing participants from 7 out of the 8 CMR programme groups. Mindfulness Teacher MK was also interviewed.

### Participant characteristics

The mean age of interview participants was 51.6 years, and most were female (73.7%, *n* = 14) and married (68.4%, *n* = 13). Most interview participants were post-transplant (57.9%, *n* = 11), from an English, Welsh, Scottish, Northern Irish or British background (68.4%, *n* = 13) and had a level 4 + education (78.9%, *n* = 15). The mean time since diagnosis was 20.42 years. An overview of interview participant characteristics is available in Table 5.

**Table 5:** Interview participant characteristics

**Characteristic**	**Participants analysed (*n* = 19)**
**Age (years)**	
n (%)	19 (100%)
Mean (SD)	51.6 (8.34)
**Gender, n (%)**	
Number with data	19 (100%)
Female	14 (73.7%)
Male	5 (26.3%)
**Ethnicity, n (%)**	
Number with data	19 (100%)
Black, African, Caribbean or Black British—African	1 (5.3%)
White—Any other White background	1 (5.3%)
Black, African, Caribbean or Black British—Caribbean	2 (10.5%)
White—English, Welsh, Scottish, Northern Irish or British	13 (68.4%)
Asian or Asian British—Indian	1 (5.3%)
White—Irish	1 (5.3%)
**Marital status, n (%)**	
Number with data	19 (100%)
Divorced	1 (10.5%)
Married	13 (68.4%)
Widowed	1 (5.3%)
Single	3 (5.3%)
**Highest educational level, n (%)**	
Number with data	19 (100%)
Level 1	1 (5.3%)
Level 3	1 (5.3%)
Level 4 +	15 (78.9%)
No qualifications	1 (5.3%)
Other	1 (5.3%)
**Years since diagnosis (years)**	
n (%)	19 (100%)
Mean (SD)	20.42 (11.33)
**Stage of CKD, n (%)**	
Number with data	19 (100%)
Post-transplant	11 (57.9%)
Stage 5	4 (21.1%)
Stage 4	4 (21.1%)

### Qualitative analysis

Three key themes with subthemes were generated (summarised with supporting quotations in Table 6); experiences of the CMR programme that facilitated subjective benefit; participants lived and shared experiences; and the experience of participating in the CMR programme. The complete qualitative findings from this study have been published [40] and a brief overview of the themes generated has been presented below.

**Table 6:** Thematic analysis themes and subthemes

Theme	Subthemes	Supporting Quotations
Experiences of the CMR programme that facilitated subjective benefit	Interest in Mindfulness and previous experience	“*I’ve done some yoga, and yoga nidra in particular, where you think your way around your body… when my kidneys first failed, whilst I was still on dialysis, I had some counselling and part of that was learning breathing techniques. So I’ve done similar things, but nothing actually called mindfulness.”* *“It was an opportunity to help me become more at peace with myself and my thoughts.”*
	Integrating Mindfulness using techniques and practices to enhance awareness and compassion	*“Mindfulness has really consolidated the fact that my feelings are valid. And that I can learn to understand them and ‘control them’. Meaning, my feelings and/or my thoughts are not necessarily… they are just that. They are things that pass through me.”* *“The practices, I have been doing ones that I found beneficial. The one that I keep going back to is the body scan, because the likes of, with the anxiety I find it’s either in my chest, with tight chest or fast beating heart, or in my stomach, it goes into knots. And that helps me calm the physical sensations of that down, and then because of that, then mentally I am calmer as well.”*
	Continuing Mindfulness practice	*“It’s really switching off from what’s going on around me. I come down to my shed in the garden, where I’ve got my office. So I can shut out the world and that’s really good. And it’s making me do it. Because I’m thinking, I’ve got to do it. Not because I have to do it, but because I want to do it.”* *“I would love refreshers. You know like you go to see the consultant every six months or something, it would be lovely to have a wee quick session just to focus your mind. Even if it was away in the distance, you would know it’s coming. I think it keeps you mindful. “*
Participants lived and shared experiences	Psychological impact of kidney disease and continuing impact of COVID-19	*“I was diagnosed and then three weeks from diagnosis I was getting dialysed. So when it’s kind of traumatic that way, and you spend all that time on dialysis away from your family, that’s a struggle.”* *“I’m still… very much so feeling the effects of COVID. Because I’ve been shielding. And I’m still semi-shielding now, so it’s been about three years. And when I feel very vulnerable… I feel like a quarter of the person I used to be.”*
	Shared experiences with other study participants	*“I would say is that the benefit we got through talking with each other in the groups, was so beneficial. So beneficial. We need more of that. Absolutely more of that. Getting that connection. Getting an understanding. That feeling that you’re not on your own.”* *“They want to talk to each other about their kidney disease. They are doing it anyway. So use those spaces. They’ve told us that’s what they are doing in the breakout rooms.”*
	Need for wellbeing support for people living with kidney disease	*“You are dealing with something at that stage, that you know your kidney is going to eventually give up, very soon probably. How do you deal with that? You’ve got a major body part that keeps your body functioning, that doesn’t work. How do you cope with that in your mind? If you can be given a key to help that along, then yeah, for sure it’s going to benefit.”* *“I felt as if I was falling at that stage, I was coming down one of them big circular slides. I just felt I was sliding and I had nothing to hold on to… mindfulness might have been better if I had it before I got an acute phase, if that makes any sense. I might have had those tools in place before, that it might have prevented those feelings of falling.”*
Practicalities of CMR programme participation	Challenges and barriers to participation	*“Life happens to these people. They become ill or they need to go into hospital. So I think it’s just anticipating that that will happen and having a plan for that.”* *“I felt that I couldn’t really say any of the bad things that I’ve experienced, because I was this sort of golden shining thing, having a successful transplant for lots of years.”*
	Experience of online delivery	*“You’re very safe in your own home. And you can mute or you can turn off the camera if you’re feeling a wee bit emotional.”* *“What I would suggest with Kidney Care UK is, they may want to provide people with iPads, that those iPads are loaned for the duration of the programme. And that person doesn’t need to do anything but just click ‘join’ and everything is there for them.”*
	Adaptations for future delivery for people living with kidney disease	*“I would have thought some sort of website with all these materials and stuff like that, and short presentations on some of the themes that the course has given, would give people an opportunity to refresh their minds.”* *“I think if you use their lived experience, experts by experience, particularly around managing, like shaky leg, managing fatigue, managing a whole host of interesting symptoms that are medicine related. Managing machines that keep you awake. I think there maybe is in some of the sessions, where you could actually just give it over to that. Because I was learning from their enquiry. So I’d say if you look at the enquiry points and tailor the questions.”*

#### Theme 1: experiences of the CMR programme that facilitated subjective benefit

The first theme explores participants’ previous experience of mindfulness and motivations to participate in the CMR programme, how participants integrated mindfulness techniques and practices into their daily lives, and their continuing mindfulness practice. Most participants stated that they were interested in participating in the programme to support their own mental wellbeing.

Participants reported that they found the programme to have been beneficial, enabling them to become more aware of their emotions, manage negative thoughts and become more compassionate towards themselves and others. Most participants were continuing to practice mindfulness and expressed an interest in an ongoing practice with other people living with kidney disease.

*“I think just the general feeling of wellbeing. When you’re doing the breathing and deep into one of the mindfulness practices… how calm and relaxing that feels and how it helps steady everything. Because that feels so good. It makes you want to carry on learning about it and to be able to do it to the best that you can.”*

Participant Identification Number CMR058

#### Theme 2: participants lived and shared experience

This theme highlights the impact of kidney disease on participants’ mental wellbeing, the value of shared experiences with other people living with kidney disease and the need for ongoing wellbeing support. Participants identified that being diagnosed with kidney disease, and subsequent treatment options and transplant procedures, had been traumatic experiences, and many experienced poor mental wellbeing, which had been exacerbated by the requirement to ‘shield’ during the COVID-19 pandemic. Most participants expressed that they particularly enjoyed connecting with other people living with kidney disease, and that shared experience was an important component of the programme.

*“More than anything, just the fact that I met people, especially when we broke off into the groups. I was actually talking to somebody who had the same disease, who was feeling the same, … it was like winning the lottery.”*

CMR024

#### Theme 3: practicalities of CMR programme participation

The final theme reflects on the practicalities of participating in the CMR programme, and potential adaptations for future delivery. Some participants experienced practical challenges to participating including illness, unforeseen hospital visits and changes to work schedules, while others found it more challenging to share their vulnerabilities with the group. Many participants enjoyed participating online, as many were continuing to reduce social interactions due to COVID-19 and expressed that they felt ‘safe’ participating online in their own home.

*“I guess I might have struggled a bit more with commitment if I’d to jump on a train and go into Edinburgh every couple of weeks. So I actually think it’s probably a useful thing. It didn’t restrict me in any way at all.”*

CMR034

Participants and the Mindfulness Teacher discussed potential adaptions that could be made to the programme for future delivery for people living with kidney disease, including the development of online resources containing video and audio presentations and practices that could be revisited by participants. Digital health interventions such as the Kidney Beam physical activity platform have been successful at improving wellbeing for people with kidney disease, providing a model that could be explored for future delivery mindfulness interventions [41].

## Discussion

This is the first study to explore the acceptability and feasibility of virtual delivery of the CMR programme for people living with chronic kidney disease. We demonstrated feasibility of recruitment to the intervention by reaching the target sample of 75 participants within one month, evidencing a high level of interest in the CMR programme within the kidney disease community. The rapid rate of recruitment may reflect the demand for psychosocial support by people living with kidney disease [10, 42], highlighted within the themes identified through the interviews. Previous research has also found that mindfulness interventions are an acceptable option for people living with chronic kidney disease [43–45], however this is the first study to demonstrate this holds true with a shortened four-week programme delivered online.

The anticipated rate of attrition was observed across the four weeks of the programme (13.3%, *n* = 10), given the mode of delivery and chronic disease population [26]; however there was an increase in attrition rate at the three-month post intervention follow up (40%, *n* = 30). Poor retention rates are an established problem in kidney disease research [46], and strategies will be implemented to improve retention rates in a definitive trial. This will include improved communication and engagement during the follow-up period with the study team, participant newsletters, explanations on the importance of collecting follow-up data, and participant incentives such as reimbursing participants for time spent completing outcome measures [47].

Study participants reported an interest in ongoing mindfulness support following the completion of the four-week CMR programme. While this may call into question whether the shortened programme is an adequate length for participants, this desire for ongoing support is also typical of longer mindfulness interventions, such as MBSR [48]. Therefore, a definitive trial is needed, with adequate follow-up, to determine whether CMR can provide sustained improvements to mental health and wellbeing, or whether additional ongoing support is needed for consistent benefit.

Improvements were observed across the clinical outcome measures, with decreases in participants’ levels of anxiety and depression, and increases in levels of self-compassion, resilience, mental wellbeing, and ability to be mindful. These findings support previous research evaluating mindfulness interventions for people with chronic kidney disease, in particular improvements in anxiety and depression [49–51], and an increased ability to be mindful [50]. However, participants emphasized that they experienced substantial benefits from the opportunity to receive social support from others who had similar lived experience. Therefore, a future randomised control trial with a larger sample size is required to rigorously evaluate the efficacy of the CMR programme, including an active control group to determine whether any effects result from the mindfulness intervention, or the provision of social support [52].

The delivery of the CMR programme online was acceptable and reflects findings from previous studies which identify alternative modes to in-person delivery, such as telephone or online, as feasible options for people living with chronic kidney disease [44, 45, 53]. Participating online was found to be acceptable in the present study due to the geographical distribution of participants across the UK and for people who are continuing to limit their social interactions due to their clinically vulnerable status.

There are study limitations to consider, notably the lack of control group, and a limited opportunity for long term follow-up with participants. Additionally, while kidney disease is more prevalent in minority ethnic groups [54], the majority of participants recruited to this study were white. The lack of diversity within mindfulness research in healthcare contexts has been recognised, as studies tend to include middle-aged, white, female participants [55], and future research should address how the CMR programme can be adapted for people living with kidney disease from ethnically diverse backgrounds to ensure the patient population is appropriately represented. Additionally, minority groups within the UK often face increased barriers to digital inclusion and may lack access to technology [56], therefore consideration should be given to the provision of tools to enable access to the CMR programme in future research.

## Conclusion

The four-week CMR programme is feasible and acceptable for people living with chronic kidney disease. Improvements in clinical outcome measures and analysis of qualitative responses suggest that the mindfulness intervention has the potential to support mental health and improve the wellbeing of this patient population. A definitive clinical trial is recommended to rigorously assess the effectiveness of the CMR programme and determine if an online mindfulness intervention is a viable solution to improve the psychological outcomes of people living with chronic kidney disease.

### Supplementary Information

**Additional file 1.** COSMIC Study CONSORT checklist. Consolidated Standards of Reporting Trial (CONSORT) extension to randomised pilot and feasibility trials 2016 checklist.

## Data Availability

The data sets for this feasibility study can be provided by contacting the authors of the paper.

## References

[1] Kidney Care UK and National Psychosocial Working Group. Psychosocial Health – A Manifesto for Action 2022. Available from: https://www.kidneycareuk.org/about-kidney-health/living-kidney-disease/mental-health/manifesto/. Accessed 01 October 2021.

[2] Donahue S, Quinn DK, Cukor D, Kimmel PL (2021). Anxiety presentations and treatments in populations with kidney disease. Semin Nephrol.

[3] Tsai Y-C, Chiu Y-W, Hung C-C, Hwang S-J, Tsai J-C, Wang S-L (2012). Association of symptoms of depression with progression of CKD. Am J Kidney Dis.

[4] Dew MA, Rosenberger EM, Myaskovsky L, Dimartini AF, Devito Dabbs AJ, Posluszny DM (2015). Depression and anxiety as risk factors for morbidity and mortality after organ transplantation. Transplantation.

[5] Murtagh FE, Addington-Hall J, Higginson IJ (2007). The prevalence of symptoms in end-stage renal disease: a systematic review. Adv Chronic Kidney Dis.

[6] Hackett ML, Jardine MJ (2017). We need to talk about depression and dialysis: but what questions should we ask, and does anyone know the answers?. Clin J Am Soc Nephrol.

[7] Seekles ML, Ormandy P, Coyne E (2019). Mapping the UK renal psychosocial workforce: the first comprehensive workforce survey. BMC Nephrol.

[8] Ortiz A, Cozzolino M, Fliser D, Fouque D, Goumenos D, Massy ZA (2021). Chronic kidney disease is a key risk factor for severe COVID-19: a call to action by the ERA-EDTA. Nephrol Dial Transplant.

[9] Antoun J, Brown DJ, Jones DJW, Sangala NC, Lewis RJ, Shepherd AI (2021). Understanding the impact of initial COVID-19 restrictions on physical activity, wellbeing and quality of life in shielding adults with end-stage renal disease in the united kingdom dialysing at home versus in-centre and their experiences with telemedicine. Int J Environ Res Public Health.

[10] McKeaveney C, Noble H, Carswell C, Johnston W, Reid J (2021). Psychosocial well-being of patients with kidney failure receiving haemodialysis during a pandemic: a survey. Healthcare.

[11] Voorend CGN, van Oevelen M, Nieberg M, Meuleman Y, Franssen CFM, Joosten H (2021). Impact of the COVID-19 pandemic on symptoms of anxiety and depression and health-related quality of life in older patients with chronic kidney disease. BMC Geriatr.

[12] American Psychological Association. Mindfulness Meditation: A Research-Proven Way to Reduce Stress 2020. Available from: https://www.apa.org/topics/mindfulness/meditation. Accessed 15 Mar 2022.

[13] Gu J, Strauss C, Bond R, Cavanagh K (2015). How do mindfulness-based cognitive therapy and mindfulness-based stress reduction improve mental health and wellbeing? A systematic review and meta-analysis of mediation studies. Clin Psychol Rev.

[14] Kabat-Zinn J (2003). Mindfulness-Based Stress Reduction (MBSR). Constructivism Human Sci.

[15] Merkes M (2010). Mindfulness-based stress reduction for people with chronic diseases. Aust J Prim Health.

[16] Kilic A, Hudson J, McCracken LM, Ruparelia R, Fawson S, Hughes LD (2021). A systematic review of the effectiveness of self-compassion-related interventions for individuals with chronic physical health conditions. Behav Ther.

[17] Razzera BN, Adamoli AN, Ranheiri MF, Oliveira MDS, Feoli AMP (2022). Impacts of mindfulness-based interventions in people undergoing hemodialysis: a systematic review. Brazilian Journal of Nephrology.

[18] Kidney Care UK. 2023. Available from: https://www.kidneycareuk.org/. Accessed 1 Sept 2021.

[19] MindfulnessUK. Compassionate Mindful Resilience 2021. Available from: https://mindfulnessuk.com/course/compassionate-mindful-resilience. Accessed 4 Sept 2021.

[20] British Association of Mindfulness-based Approaches 2023. Available from: https://bamba.org.uk/. Accessed 15 Mar 2022.

[21] Atkinson K. Compassionate Mindful Inquiry in Therapeutic Practice. London, UK: Singing Dragon; 2020.

[22] Wilson A, McKeaveney C, Carswell C, Atkinson K, Burton S, McVeigh C (2022). Examining the acceptability and feasibility of the Compassionate Mindful Resilience (CMR) programme in adult patients with chronic kidney disease: the COSMIC study protocol. Healthcare.

[23] Kounali D, Button KS, Lewis G, Gilbody S, Kessler D, Araya R (2022). How much change is enough? Evidence from a longitudinal study on depression in UK primary care. Psychol Med.

[24] Spitzer RL, Kroenke K, Williams JBW, Löwe B (2006). A brief measure for assessing generalized anxiety disorder. Arch Intern Med.

[25] Stoop CH, Nefs G, Pommer AM, Pop VJ, Pouwer F (2015). Effectiveness of a stepped care intervention for anxiety and depression in people with diabetes, asthma or COPD in primary care: A randomized controlled trial. J Affect Disord.

[26] Meyerowitz-Katz G, Ravi S, Arnolda L, Feng X, Maberly G, Astell-Burt T (2020). Rates of attrition and dropout in app-based interventions for chronic disease: systematic review and meta-analysis. J Med Internet Res.

[27] Silver Lake. Qualtrics. Provo, Utah, USA2020.

[28] Kroenke K, Spitzer RL, Williams JBW (2001). The PHQ-9. J Gen Intern Med.

[29] Raes F, Pommier E, Neff KD, Van Gucht D (2011). Construction and factorial validation of a short form of the Self-Compassion Scale. Clin Psychol Psychother.

[30] Baer RA, Smith GT, Lykins E, Button D, Krietemeyer J, Sauer S (2008). Construct validity of the five facet mindfulness questionnaire in meditating and nonmeditating samples. Assessment.

[31] Tennant R, Hiller L, Fishwick R, Platt S, Joseph S, Weich S (2007). The Warwick-Edinburgh Mental Well-being Scale (WEMWBS): development and UK validation. Health Qual Life Outcomes.

[32] Perry JL, Clough PJ, Crust L, Earle K, Nicholls AR (2013). Factorial validity of the mental toughness questionnaire-48. Personality Individ Differ.

[33] IBM. SPSS Statistics for Windows. Armonk, NY: IBM Corp; 2021.

[34] Polkinghorne DE (2005). Language and meaning: Data collection in qualitative research. J Couns Psychol.

[35] Guest G, Bunce A, Johnson L (2006). How many interviews are enough?: an experiment with data saturation and variability. Field Methods.

[36] Forman J, Heisler M, Damschroder LJ, Kaselitz E, Kerr EA (2017). Development and application of the RE-AIM QuEST mixed methods framework for program evaluation. Prev Med Rep.

[37] Archibald MM, Ambagtsheer RC, Casey MG, Lawless M (2019). Using zoom videoconferencing for qualitative data collection: perceptions and experiences of researchers and participants. Int J Qual Methods.

[38] Lumivero. NVivo, Version 12. QSR International Pty Ltd; 2018.

[39] Braun V, Clarke V (2006). Using thematic analysis in psychology. Qual Res Psychol.

[40] Wilson A, McKeaveney C, Carswell C, Atkinson K, Burton S, McVeigh C (2023). Experiences of people with kidney disease following the implementation of the compassionate mindful resilience programme: qualitative findings from the COSMIC study. Healthcare.

[41] Greenwood SA, Young HML, Briggs J, Castle EM, Walklin C, Haggis L, et al. Evaluating the effect of a digital health intervention to enhance physical activity in people with chronic kidney disease (Kidney BEAM): a multicentre, randomised controlled trial in the UK. The Lancet Digital Health. 2023. 10.1016/S2589-7500(23)00204-2 37968170

[42] Seekles MK, Coyne, E. Ormandy, P. Wells, L. Bevin A. & Danbury-Lee, A. The UK renal psychosocial workforce - a mapping exercise. British Renal Society & Kidney Care UK workforce report, University of Salford.; 2018.

[43] Thomas Z, Novak M, Platas SGT, Gautier M, Holgin AP, Fox R (2017). Brief mindfulness meditation for depression and anxiety symptoms in patients undergoing hemodialysis: a pilot feasibility study. Clin J Am Soc Nephrol.

[44] Carver JA, Cheung KL (2021). Feasibility and acceptability of a yogic breathing/mindfulness meditation e-intervention on symptoms and COVID-19-associated anxiety in patients receiving dialysis. J Palliat Med.

[45] Hernandez R, Burrows B, Wilund K, Cohn M, Xu S, Moskowitz JT (2018). Feasibility of an Internet-based positive psychological intervention for hemodialysis patients with symptoms of depression. Soc Work Health Care.

[46] Natale P, Gutman T, Howell M, Dansie K, Hawley CM, Cho Y (2020). Recruitment and retention in clinical trials in chronic kidney disease: report from national workshops with patients, caregivers and health professionals. Nephrol Dial Transplant.

[47] Gillies K, Kearney A, Keenan C, Treweek S, Hudson J, Brueton VC (2021). Strategies to improve retention in randomised trials. Cochrane Database Syst Rev.

[48] Tulloh RMR, Garratt V, Tagney J, Turner-Cobb J, Marques E, Greenwood R (2018). A pilot randomised controlled trial investigating a mindfulness-based stress reduction (MBSR) intervention in individuals with pulmonary arterial hypertension (PAH): the PATHWAYS study. Pilot Feasib Stud.

[49] Nassim M, Park H, Dikaios E, Potes A, Elbaz S, Mc Veigh C (2021). Brief mindfulness intervention vs. health enhancement program for patients undergoing dialysis: a randomized controlled trial. Healthcare (Basel).

[50] Alhawatmeh H, Alshammari S, Rababah JA (2022). Effects of mindfulness meditation on trait mindfulness, perceived stress, emotion regulation, and quality of life in hemodialysis patients: a randomized controlled trial. Int J Nurs Sci.

[51] Assarian F (2021). Efficacy of mindfulness-based stress reduction in hemodialysis patients with anxiety and depression: a randomized, double-blind, parallel-group trial. Electron Physician.

[52] Bennett PN, Ngo T, Kalife C, Schiller B (2018). Improving wellbeing in patients undergoing dialysis: can meditation help?. Semin Dial.

[53] Gross CR, Reilly-Spong M, Park T, Zhao R, Gurvich OV, Ibrahim HN (2017). Telephone-adapted Mindfulness-based Stress Reduction (tMBSR) for patients awaiting kidney transplantation. Contemp Clin Trials.

[54] Norris KC, Agodoa LY (2005). Unraveling the racial disparities associated with kidney disease. Kidney Int.

[55] Chin G, Anyanso V, Greeson J (2019). Addressing diversity in mindfulness research on health: a narrative review using the addressing framework. Cooper Rowan Med J.

[56] Poole L, Ramasawmy M, Banerjee A (2021). Digital first during the COVID-19 pandemic: does ethnicity matter?. Lancet Public Health.

